# Computational Modeling and Imaging of the Intracellular Oxygen Gradient

**DOI:** 10.3390/ijms232012597

**Published:** 2022-10-20

**Authors:** Andrew J. H. Sedlack, Rozhin Penjweini, Katie A. Link, Alexandra Brown, Jeonghan Kim, Sung-Jun Park, Jay H. Chung, Nicole Y. Morgan, Jay R. Knutson

**Affiliations:** 1 Biomedical Engineering and Physical Science Shared Resource, National Institute of Biomedical Imaging and Bioengineering (NIBIB), National Institutes of Health (NIH), Bethesda, MD 20892-5766, USA; 2 Laboratory of Advanced Microscopy and Biophotonics, National Heart, Lung, and Blood Institute (NHLBI), National Institutes of Health (NIH), Bethesda, MD 20892-1412, USA; 3 Laboratory of Obesity and Aging Research, National Heart, Lung, and Blood Institute (NHLBI), National Institutes of Health (NIH), Bethesda, MD 20892-1412, USA; 4 Department of Biochemistry, College of Medicine, The Catholic University of Korea, Seoul 06591, Korea

**Keywords:** myoglobin-mCherry, mitochondrial pO_2_ gradient, finite-element analysis

## Abstract

Computational modeling can provide a mechanistic and quantitative framework for describing intracellular spatial heterogeneity of solutes such as oxygen partial pressure (pO_2_). This study develops and evaluates a finite-element model of oxygen-consuming mitochondrial bioenergetics using the COMSOL Multiphysics program. The model derives steady-state oxygen (O_2_) distributions from Fickian diffusion and Michaelis–Menten consumption kinetics in the mitochondria and cytoplasm. Intrinsic model parameters such as diffusivity and maximum consumption rate were estimated from previously published values for isolated and intact mitochondria. The model was compared with experimental data collected for the intracellular and mitochondrial pO_2_ levels in human cervical cancer cells (HeLa) in different respiratory states and under different levels of imposed pO_2_. Experimental pO_2_ gradients were measured using lifetime imaging of a Förster resonance energy transfer (FRET)-based O_2_ sensor, Myoglobin-mCherry, which offers in situ real-time and noninvasive measurements of subcellular pO_2_ in living cells. On the basis of these results, the model qualitatively predicted (1) the integrated experimental data from mitochondria under diverse experimental conditions, and (2) the impact of changes in one or more mitochondrial processes on overall bioenergetics.

## 1. Introduction

It is widely recognized that intracellular oxygen partial pressure (pO_2_) plays an important role in cellular function and metabolism. In most functioning cells, the oxygen (O_2_) level needs to be sufficient to maintain mitochondrial activity, providing adenosine triphosphate (ATP) and other substances essential for normal cell function [1,2]. Despite broad consensus on the importance of O_2_ concentration ([O_2_]), many important quantitative aspects of the physiological and pathophysiological phenomena related to pO_2_ remain unclear. This is due, in part, to a lack of methodology to make accurate measurements of intracellular and subcellular pO_2_.

In extant experimental and theoretical work involving the role of pO_2_, the values used are frequently imposed extracellular pO_2_, because those values can be more readily measured. This approach is based on the assumption that there are no steep pO_2_ gradients in or around cells. While there is ample computational and experimental precedent stating that sharp gradients over the span of a few microns are unlikely, regional gradients within and around cells have been measured, and their presence is supported by some models [3,4,5,6]. The mechanism of these pO_2_ gradients, however, remains uncertain due to several untested key assumptions. The role of lipid bilayers (such as the cell membrane and intracellular compartmental membranes) as barriers to gas diffusion is highly controversial, with varying results having been measured or computed depending on the precise composition of the membrane [3,7,8,9,10,11].

Early modelers predicted that regional O_2_ gradients across or within cells were not possible within diffusion-dominated regimes [4,12]. These models continue to be cited as evidence that there are limited, if any, intracellular O_2_ gradients. Significantly, these models only considered O_2_ gradients arising outside a mitochondrion or cell sitting in a 3D O_2_ permeable volume, such as a cell suspension or within permeable tissue; the geometry used for adherent tissue culture, in which cells sit on an O_2_-impermeable substrate, was not considered [13,14,15].

While early modeling results led to a reconsideration of then-available measurement techniques, significant O_2_ gradients across cells have continued to be observed from electron paramagnetic resonance (EPR) spectroscopy and more modern phosphorescence and fluorescence lifetime and intensity-based imaging (using either a microplate reader or a microscope) [6,16,17,18,19]. Some studies showed significant intracellular gradients purely on the basis of differences in mean compartmental average values rather than imaging across whole cells [5,20]. The modeling of local gradients formed on length scales comparable to the subcellular structure of mitochondrial organization is made more tractable with finite-element methods. This recently enabled full-cell bioenergetic finite-element models of mitochondrial O_2_ consumption in cardiomyocytes, which showed gradients as steep as 2 Torr/μm [21,22]. We now present a model for subcellular O_2_ concentration and consumption under common in vitro conditions to better complement current imaging and measurement techniques.

Most recently, we used fluorescence lifetime imaging (FLIM) of a Förster resonance energy transfer (FRET)-based sensor, Myoglobin (Myo)-mCherry, with targeting sequences attached that enabled us to measure pO_2_ within the mitochondria and cytosol, finding differences of up to 11 mmHg between the compartments under some conditions [23,24]. Using microscopic images of mitochondria in C2C12 mouse myoblast cells, we developed a model that incorporated an extended, spatially complex mitochondrial compartment in which O_2_ consumption is governed by Michaelis–Menten kinetics, and we looked at the resulting gradients in subcellular [O_2_] in and around this compartment. Looking at a range of experimental conditions and consensus values from the literature for O_2_ diffusion, solubility, and consumption, we saw qualitative agreement between the computational model and experimental measurements of the differences (0.4, 1, 3, and 4 mmHg at applied [O_2_] of 0.5%, 5%, 10%, and 18.5%, respectively) between the intracellular and mitochondrial compartments in a human cervical cancer cell line (HeLa).

## 2. Results

### 2.1. Measurements of the Intracellular pO_2_ Gradients

In tissue culture of adherent cells, the balance between mitochondrial O_2_ consumption of the cell monolayer at the bottom of the petri dish and the primarily diffusive transport of O_2_ through media is a major determinant of pericellular O_2_. For dense layers of metabolically active cells, pericellular pO_2_ can drop quite low, even in ambient atmospheric [O_2_]. Cells with low or non-consuming mitochondria, on the other hand, are likely to experience greater intracellular pO_2_ than those more rapidly consuming O_2_ [25].

**Figure 1:**
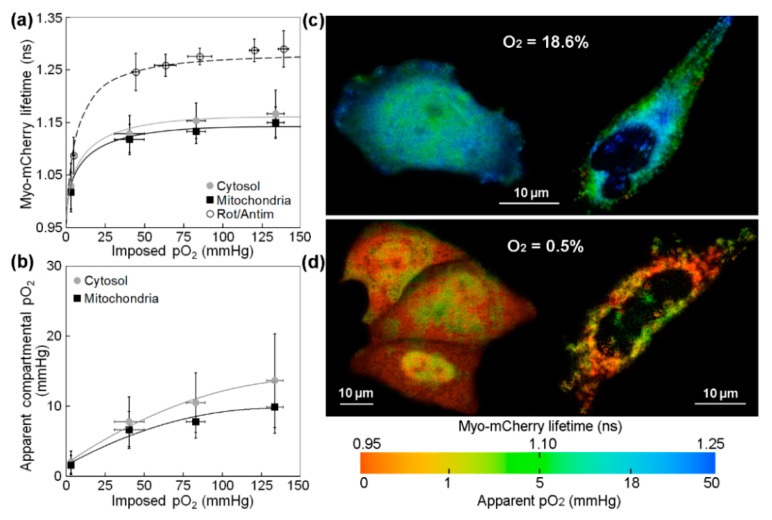
(**a**) Average fluorescence lifetime of Myo-mCherry in the cytosol and mitochondria of respiring and non-respiring (rotenone/antimycin treated) HeLa cells plotted versus medium pO_2_ values obtained from Oxylite Pro measurements; the data are shown with the best hyperbolic fit obtained from Equation (1). (**b**) The apparent intracellular pO_2_ versus the medium imposed pO_2_; error bars are the standard deviations. Pseudocolor mapping of Myo-mCherry lifetime (or pO_2_) in the cytosol (left) and mitochondria (right) of HeLa cells at atmospheric pO_2_ of (**c**) 18.6% and (**d**) 0.5%. In the color bars, red indicates lower lifetime and pO_2_ values, whereas blue indicates higher values.

Figure 1a shows intracellular and mitochondrial pO_2_ at different values of imposed pO_2_, as measured using lifetime imaging of Myo-mCherry and mitochondrial-targeted Myo-mCherry (mtMyo-mCherry) and a calibration curve derived from non-respiring (rotenone/antimycin-treated) cells. The differences between the average lifetime values obtained for the non-respiring cells transfected with Myo-mCherry or mtMyo-mCherry were not statistically significant; the data shown for non-respiring cells in Figure 1a are for cells transfected with Myo-mCherry. Here, we use imposed pO_2_ to mean the extracellular [O_2_] measured using the OxyLite Pro device; this fiber probe quantifies pO_2_ in the medium, approximately 100 microns above the cell layer. The lifetime data were fitted using the hyperbolic equation Equation (1) (see Section 4.6); fitting parameters are available in Table 1. Rearranging Equation (1) and using the values of *K* and *τ_max_* obtained for the rotenone/antimycin-treated cells, we obtained a calibration curve relating the lifetime of the Myo-mCherry probes to pO_2_. Using this calibration, we calculate the effective pO_2_ for each subcellular compartment in respiring cells from the lifetime data, and we plot it as a function of imposed pO_2_ in Figure 1b. Pseudocolor mapping examples of pO_2_ in the intracellular and mitochondrial environments at normoxia and hypoxia are plotted in Figure 1c,d, respectively. More detailed descriptions of the pO_2_ mapping procedure can be found in Ref. [23].

By examining a variety of anoxic through normoxic conditions, one can see that the intracellular versus imposed pO_2_ trends are similar and hyperbolic for all cell compartments. Clearly, the sustained intracellular pO_2_ levels, well below those applied, reflect O_2_ consumption in HeLa cells. For the highest atmospheric [O_2_] used here (18.6%, or about 140 mmHg), we see apparent intracellular pO_2_ levels of 14 mmHg in the cytosol and 10 mmHg in the mitochondria.

Note that the medium-imposed pO_2_ measured with the OxyLite is assumed to be identical to that in the intracellular volume only for the non-respiring cells; these values are clearly different for cells with active mitochondrial respiration. The myoglobin saturation-controlled lifetime values still represent in situ metabolic pO_2_. In other words, the affinity for O_2_ of the myoglobin in the chimeric probe defines the relationship between lifetime and local pO_2_. The only assumption in this back-calculation mapping is that rotenone/antimycin joint treatment effectively equalizes medium-imposed and internal local levels.

### 2.2. COMSOL Modeling of the Intracellular pO_2_ Gradients

**Figure 2:**
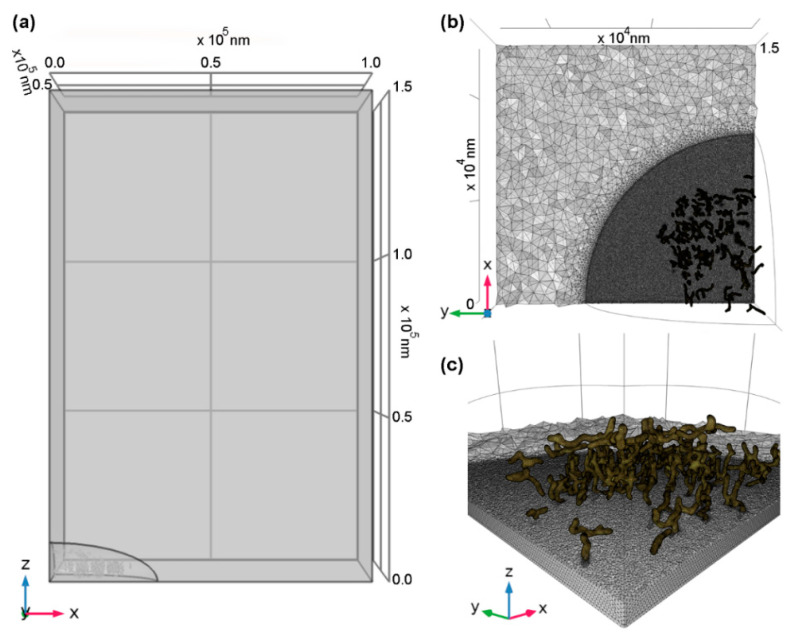
(**a**) Model geometry viewed in profile; (**b**) cross-section of mesh viewed from above and (**c**) center. Rectangular volume defines a column of medium with eighth section of an ellipsoid defining the cytoplasm and live-cell imaged mitochondrial geometry (in gold for meshes). Boundary conditions are as described in Section 4.

We modeled [O_2_] at equilibrium in the diffusion-dominated regime surrounding cells grown in monolayer culture. As shown in Figure 2, we used a symmetric model representative of a uniform square grid of cells, specifically, a periodic boundary condition using one-quarter of a single cell in a rectangular column of medium with symmetric (i.e., zero flux) lateral boundaries. Mitochondrial structure for the model was scanned from a C2C12 mouse myoblast cell (see Figure S1 in Supplementary Materials) in order to provide insight into the possible impact of the spatial structure and organization of the mitochondrial volume on subcellular O_2_ gradients. We adjusted the edge length of the medium column (i.e., distance between cells) to represent different cellular densities (i.e., confluence level), and a constant applied [O_2_] was assumed at the top boundary.

The equilibrium intracellular pO_2_ gradients were calculated for a range of applied [O_2_], cell spacings, and lower boundary conditions. Models were also generated for different maximal mitochondrial O_2_ consumption rates and diffusion conditions; however, in the end, only the highest consumption rate seen in the literature and consensus diffusion constants were used here. The resulting calculations for mean pO_2_ in mitochondrial and cytosolic compartments are presented in Table 2. Alternatively, minimal mitochondrial and maximal cytoplasmic values are presented in Supplementary Table S1 in order to show the maximal difference observed between compartments.

As in the experimental measurements on substrates with low O_2_ permeability, the difference between the mitochondrial and cytosolic compartments is significantly smaller at the lowest values for extracellular pO_2_. At higher (5–20%) applied O_2_ concentrations, the maximal difference is 2.4–2.7 mmHg for the cells plated on glass or Ibidi polymer coverslips, similar to the differences seen experimentally with FLIM-FRET in Figure 1. However, the calculated values for intracellular O_2_ concentrations are substantially higher than expected if we assume the [O_2_] at the top of the model volume is identical to the imposed O_2_ measured with the OxyLite Pro. Looking at the model results for 10% applied O_2_ and cells growing on glass with a 70 µm spacing, we see cytoplasmic [O_2_] of 44.6 mmHg in the model, compared to 10.5 mmHg found for the 10% imposed O_2_ point in Figure 1b.

**Figure 3:**
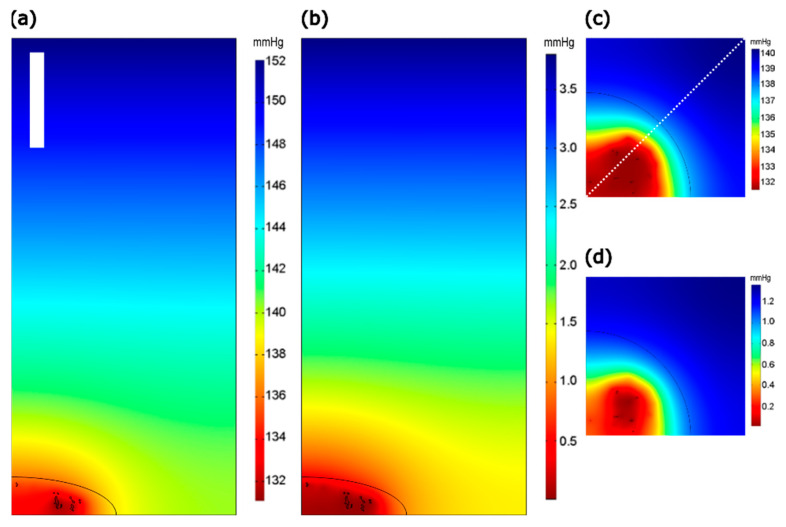
The pO_2_ (mmHg) slices of model with 100 μm cell–cell spacing and glass lower boundary at equilibrium with applied pO_2_ of (**a**,**c**) 20% and (**b**,**d**) 0.5%. (**a**,**b**) Planes through y = x (i.e., diagonal to the square grid, in order to show longer dimension—shown as dotted line in panel (**c**)) and (**c**,**d**) xy planes through z = 1 μm. Color scales are fitted to individual panel ranges. All panels are the same geometric scale; the scalebar in (**a**) is 30 μm.

Slices showing pO_2_ gradients at equilibrium for 100 μm cell–cell spacing are presented in Figure 3. At lower applied O_2_ concentrations, pericellular levels drop below the $K_{M}$ of mitochondrial consumption (0.078 mmHg), and diffusion cannot replace O_2_ as rapidly as it is being consumed by mitochondria, giving rise to a region of relatively uniform hypoxia around the mitochondria. At higher applied O_2_ levels, there is a greater amount of O_2_ available in adjacent regions to replenish the consuming regions; hence, the gradient does not ‘bottom out’ in this way.

**Figure 4:**
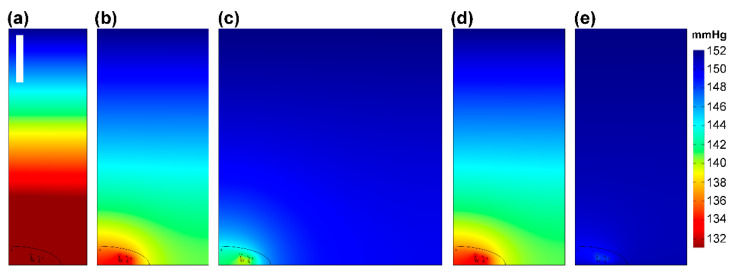
Confluence and substrate effects. The pO_2_ (mmHg) slices of model with glass lower boundary at equilibrium with cell spacing of (**a**) 70 μm, (**b**) 100 μm, and (**c**) 200 um, and with 100 μm spacing but lower boundary of (**d**) Ibidi polymer or (**e**) 200 μm PDMS, each over atmosphere. Planes through y = x (i.e., diagonal to the square grid, in order to show longer dimension, shown by dotted line in Figure 3a). All panels are the same geometric scale; the scalebar in (**a**) is 30 μm. All panels are in the same color scale, as per bar at right.

Figure 4a–c compare O_2_ gradients in models with varying cell densities. Center-to-center cell spacings of 70, 100, and 200 μm correspond to cell seeding densities of 2.4 × 10^4^, 1 × 10^4^, 2.5 × 10^3^, and 1.1 × 10^3^ cells/cm^2^. At cell spacings at or beyond 200 μm, O_2_ levels at the substrate in between cells reached those at the top surface of the computational volume 150 μm above the cell substrate, as expected. At cell spacings less than 200 μm, the monolayer significantly depleted the amount of O_2_ at the bottom of the model, resulting in significant gradients in z across the full computational volume. Spacing of 300 μm was very similar to 200 μm. Figure 4d–e shows the model in cases with O_2_ supply from below as well as above, at a limited rate through Ibidi polymer slides in Figure 4d and at a more significant rate through a 200 μm thick layer of polydimethylsiloxane (PDMS) in Figure 4e. Critically comparing Figure 4b,e shows that, with O_2_ supply arriving from multiple directions, the lateral and axial O_2_ gradients are substantially reduced relative to the case with cells against glass (with O_2_ arriving only from above), in both the intracellular and the extracellular spaces.

## 3. Discussion

The model shows that, as seen in vitro, larger gradients are formed by cells against O_2_-impermeable substrates vs. those in suspension or on O_2_-permeable substrates. The computational results showing steep gradients in z and O_2_ depletion, especially pronounced at lower applied O_2_ levels, support the idea that cells growing on O_2_-impermeable surfaces are likely to deplete O_2_ levels in their vicinity much more so than earlier models of cells in suspension predicted [3]. This is in line with the experimental results seen here, as well as published work on modeling O_2_ delivery in tissue culture which has seen O_2_ as a limiting factor in high-density monolayer culture [26].

The model and measurements both show [O_2_] in the mitochondrial compartment to be lower than that in the cytosol, with this difference decreasing in magnitude at the lowest ambient concentrations. Furthermore, the difference in pO_2_ between mitochondrial and cytosolic compartments is relatively consistent between the experiment (1, 3, and 4 mmHg at applied [O_2_] of 5%, 10%, and 18.6%, respectively) and the model (up to 2.4 and 2.7 mmHg at applied [O_2_] of 5% and 20%), and relatively stable for the model at all but the lowest applied [O_2_] of 0.5% (0.4 mmHg based on the measurements and 0.4–0.8 mmHg based on the model). However, the intracellular O_2_ concentrations measured with FLIM-FRET for higher values of imposed O_2_ are substantially lower than those found for the same value of applied O_2_ in the model. For example, intracellular O_2_ found in the model for a 70 μm cell spacing and an applied [O_2_] of 10% is three times as high as that seen for experimental measurements with imposed pO_2_ of 18.6%, although the intracellular O_2_ found at 5% applied [O_2_] is consistent between measurement and model. To confirm whether the measured intracellular pO_2_ levels are plausible, the expression of hypoxia-inducible factor (HIF)-1α in HeLa cells was investigated at all applied O_2_%. On the basis of the results shown in Figure S2 (in Supplementary Materials), HIF-1α was detected at all imposed O_2_%, even at 18.6%. The fact that we see essentially the same HIF-1α expression level at 5% and hypoxia (when it should be less at 5%) means that the measured low intracellular pO_2_ levels are indeed plausible [27]. These large differences between the absolute magnitude of [O_2_] in the experiments and model can likely be reconciled by considering how the imposed pO_2_ measured with the OxyLite relates to the applied pO_2_ fixed boundary used in the model calculations; the assumption that they are identical is almost certainly incorrect. This could arise from experimental factors, including uncertainty about the sampling volume of the OxyLite probe or nonuniformity of the cell layer, or from oversimplification in the model definition, particularly the omission of convective transport in the medium. The implications of this in interpreting the model are discussed in more detail in Section 3.3.

In addition, there are several factors we considered which might explain why the experimentally observed differences between pO_2_ in mitochondrial and cytosolic compartments are slightly larger than those calculated in the model at higher applied [O_2_]: (i) the observation of a gradient in pO_2_ between the compartments partly arises from the presence of an *extracellular* O_2_ gradient in the z direction; (ii) the potential localization of the FLIM-FRET probe through mitophagy within some unexpected compartment like lysosomes, where effects of the lysosomal refractive index and pH on the lifetime values obtained for Myo-mCherry may be confounding; (iii) other factors not considered in the model (e.g., active transport; significantly higher than expected barriers to O_2_ diffusion in the membrane, etc.). Targeting of the indicator to compartments is not geometrically precise like the model. Since the periphery of the cell (further away from the center of mitochondrial mass) has higher pO_2_, another critical parameter in understanding the difference between mean ‘mitochondrial’ and ‘cytosolic’ [O_2_] is the mean distance beyond the extent of the central mitochondrial network (horizontally or vertically) that the ‘cytosol’ extends.

### 3.1. Effects of the Mitochondrial O_2_ Consumption and Intracellular pO_2_ Gradient in z

The scientific community generally accepts that cell culture is not a perfect model system. Three factors (O_2_ above the medium, dissolved O_2_ in the medium, and O_2_ consumption by the cells) interact to create an [O_2_] gradient from the surface of the medium down to the surface of the cells [26]. This concentration gradient is the force that drives the flux of O_2_ through the medium to the underlying cells. Modification of any of the above factors alters the gradient and, therefore, the rate of O_2_ delivery to the cells [26].

**Figure 5:**
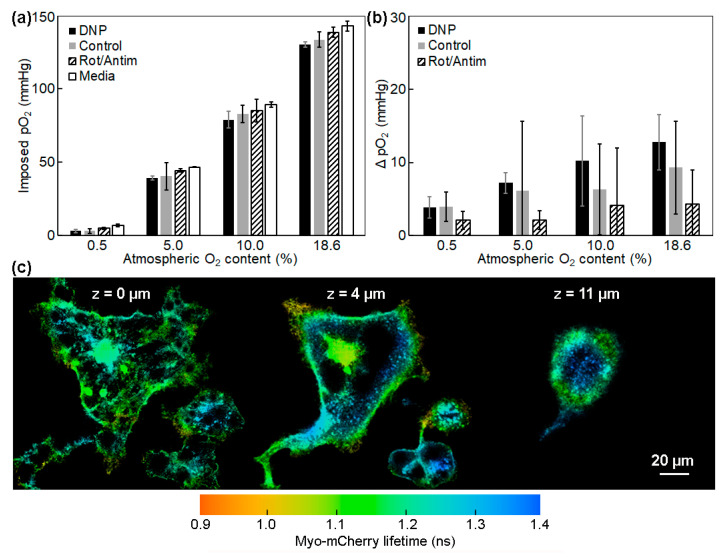
(**a**) The imposed pO_2_ measured with OxyLite Pro in culture medium with or without respiring HeLa cells; the x axis represents the applied atmospheric O_2_% in the stage incubator. (**b**) Change in O_2_ concentration (ΔpO_2_) measured with the OxyLite as a function of cellular respiration and applied pO_2_, relative to values obtained for the medium (without cells). The error bars in (**a**,**b**) are the standard deviations. (**c**) Pseudocolor mapping of Myo-mCherry fluorescence lifetime in the plasma membrane of HeLa cells at different heights above the substrate. The cells were exposed to atmospheric [O_2_] of 18.6%.

The mitochondrial O_2_ consumption of the cell monolayer at the bottom of the Petri dish is a major determinant of pericellular [O_2_] and, hence, the steepness of the O_2_ gradient in the medium covering the cells [26]. To investigate this phenomenon, we used the OxyLite bare fiber probe to measure the imposed pO_2_ (in mmHg) above the cell monolayer with and without respiring cells present. As shown in Figure 5a, mitochondrial O_2_ consumption influenced the imposed pO_2_ and, as a consequence, culture medium (without cells) and cells treated with rotenone/antimycin (non-consuming mitochondria) showed higher imposed pO_2_ measurements than respiring cells [25]. We further explored the effect of ramping up mitochondrial O_2_ consumption on the imposed pO_2_ measurement by adding the uncoupler DNP, which causes cells to consume O_2_ at a heightened rate, and we found a further reduction in the imposed pO_2_ value measured with the OxyLite. Figure 5b shows the difference in measured pO_2_ (ΔpO_2_) as a function of cellular respiration and atmospheric [O_2_], obtained by subtracting the pO_2_ values with cells present from those obtained for the medium (without cells). Please note that the OxyLite sensing volume is a porous hemisphere ~250 microns in diameter affixed to the fiber tip that measures the average medium pO_2_ at levels everywhere from the dish surface to ~125 µm above, with average z distance of about 100 μm.

It is reported that the respiratory rate in cellular system is under enzymatic control over a wide range of pO_2_ [28], and the question of whether or not the membrane is a barrier to O_2_ transport can be relevant only at low concentrations. The question of the relative magnitudes of diffusion limitations in the surrounding medium and in the cell membrane is still unanswered. Herein, the gradient steepness between the medium and intracellular compartment was preliminarily evaluated with an external plasma membrane version of Myo-mCherry (pmMyo-mCherry). Lifetime imaging of pmMyo-mCherry was performed at various depths (z) to see the possible intracellular O_2_ gradient in that direction. As shown in Figure 5c, the mean lifetime of pmMyo-mCherry was measured to be 1.18 ns in the basal plasma membrane, and there was a maximum of 6% lifetime increase when we moved 11 µm toward top of the cell surface; pO_2_ changed from ~13.5 mmHg at basal plasma membrane to 47.7 mmHg at the top of the cell surface. Although further characterization of this probe is needed, it may be helpful in quantifying gradients across the cell and in assessing transport limitations that might arise from the membrane itself.

### 3.2. The Potential Effects of pH and Refractive Index on the Lifetime Values of Myo-mCherry

**Figure 6:**
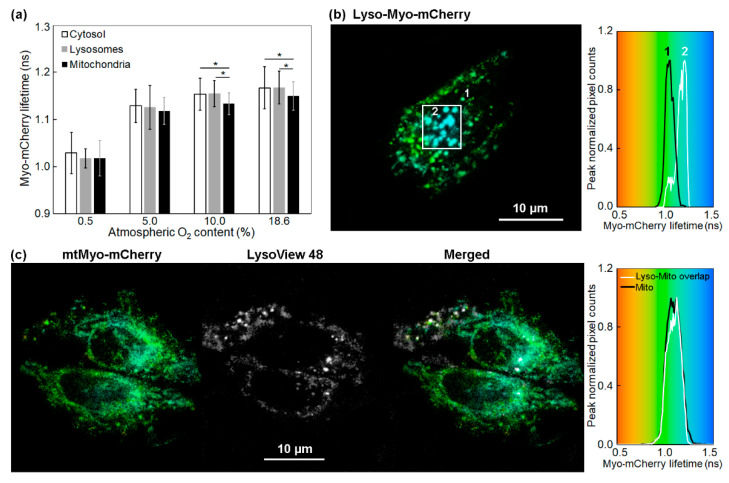
The changes of Myo-mCherry lifetime in HeLa cells using probes targeted to different subcellular compartments. (**a**) A comparison of the average fluorescence lifetime of Myo-mCherry in cytosol, lysosomes, and mitochondria; cells were exposed to external O_2_% from 18.6% to 0.5%. The error bars are the standard deviations. * *p* < 0.05. (**b**) Pseudocolor mapping and distribution histogram of Lyso-Myo-mCherry fluorescence lifetime in a HeLa cell at 5% atmospheric [O_2_]. (**c**) Pseudocolor mapping and distribution histogram of mtMyo-mCherry fluorescence lifetime in the mitochondria-lysosome contact environment.

Due to many advances in methodology, the pH value and effective refractive index of cellular compartments have become widely known. In live HeLa cells, the pH values of the nucleus, cytoplasm, and mitochondria are reported to be 5.6, 7.1, and 7.9, respectively [29,30,31]. Lysosomes generate an acidic environment of pH 4.5–5.0 and accommodate hydrolytic enzymes to degrade engulfed biomolecules. Lysosomes also have the largest refractive index among the cellular organelles. In live HeLa cells suspended in culture medium, the refractive index measured by tomographic phase imaging was reported to be 1.355–1.365 RIU for the nucleus, 1.360–1.390 RIU for the cytoplasm, 1.400–1.420 RIU for the mitochondria, and 1.600 RIU for the lysosomes [32]. The inverse lifetime of green fluorescent protein (GFP) is a function of the refractive index squared of their environment [32]. Herein, we examined the possibility of either the degradation of Myo-mCherry in the lysosomes or lifetime change in a higher refractive index environment. Figure 6a compares the average lifetime values of Myo-mCherry in the cytosol of the HeLa cells with those in their lysosomes and mitochondria at different imposed O_2_%. According to the Mann–Whitney U test, there was no statistically significant difference between the lifetime values obtained for lysosomes and cytosol (*p* ≥ 0.60). Figure 6b shows the lifetime distribution of Myo-mCherry in the lysosomes of a typical HeLa cell at an atmospheric [O_2_] of 5%. Lysosomal targeting did not shorten the lifetime values as expected vs. cytosol; in fact, a pronounced increase of the Myo-mCherry lifetime (~0.15 ns) is sometimes seen in some cells (region specified with a white square). However, this feature was only present in a small subpopulation of the HeLa cells.

Lastly, the effect of the higher lysosomal refractive index and lower pH on the lifetime values of Myo-mCherry in mitochondria–lysosome contact sites was also investigated. Mitochondria–lysosome contact sites were recently identified as inter-organelle membrane contacts involving the dynamic tethering of mitochondria with lysosomes. Importantly, mitochondria–lysosome contacts allow for the bidirectional regulation of both mitochondrial and lysosomal network dynamics, and they mediate their direct interaction in a pathway distinct from mitophagy or lysosomal degradation of mitochondrial-derived vesicles [33]. As shown in Figure 6c, the cells transfected with mtMyo-mCherry were labelled with LysoView 488 and changes in the lifetime of mtMyo-mCherry in the cross-correlated regions were inspected. Although, from 30% to 60% of the mitochondria overlapped with lysosomes, we did not see any significant difference between the lifetime in the cross-correlated regions and that in the whole cell.

### 3.3. Simplifications of O_2_ Transport in Media

The computational model presented here is aimed at generally understanding intracellular O_2_ concentrations and gradients, rather than predicting precise pericellular O_2_ concentrations as a function of experimental conditions. Each calculation was performed with a fixed [O_2_] at the upper boundary 150 μm above the cell substrate, and only considered diffusive transport in the medium. In the experimental setup, the [O_2_] at this position depends heavily on convective and diffusive transport through the ~3 mm medium layer from the atmospheric supply to the O_2_-consuming monolayer. Since consumption is only heterogeneous at the monolayer, the z-gradient caused by consumption is effectively uniform in x and y beyond a short distance from the lower boundary. Hence, the applied O_2_ used in the model should not be directly compared to the atmospheric supply used experimentally, but rather understood as a simplification of a region where [O_2_] is dictated by the experimental factors above. Values for these applied O_2_ concentrations were chosen to provide a variety of conditions, representative of the extracellular conditions measured experimentally.

As discussed above in Section 3.1, the OxyLite probe samples approximately a 250 μm hemisphere oriented downward, as close to the cell monolayer as possible without touching, expected to be approximately 125 μm above. At a first glance, it would seem that the OxyLite measurement of the imposed pO_2_ should be reasonably well-matched to the upper boundary condition used in the computational model. However, there are a number of complicating factors and uncertainties.

First, since the OxyLite samples from an extended volume, the correspondence between the measured pO_2_ values and the actual value at 150 μm above the substrate depends on the nature of the O_2_ gradient in that region. It would not be surprising if the [O_2_] measured with the OxyLite was substantially higher than the immediate pericellular [O_2_] for experiments with active cellular respiration, particularly at higher levels of atmospheric O_2_. Due to the uncertainty in measurement depth for the OxyLite, it is likely that the model boundary [O_2_] that best matches the experimental conditions is lower than the extracellular concentration measured by the OxyLite. Second, the OxyLite measurements were taken over a culture area some distance from the imaging region, in order to avoid damaging the probe with the two-photon pulsed laser. It is possible that the density of the monolayer (and hence its O_2_ consumption) could differ between these two regions. Third, cells in the experiments were significantly more confluent (phase contrast images of the cell confluency in Figure S3 in Supplementary Materials) than the modeled cells. While not expected to significantly affect the intracellular gradients seen, this *would* have a significant impact on the gradient in z and on the pericellular [O_2_]. Fourth, there is convective transport in the medium, and it is possible that this is the dominant transport mechanism at least through the upper parts of the 3 mm medium layer. The relative size of contributions from convection and diffusion in this experimental setup over the much smaller distances considered in the computational model is an open question, but it is likely that convection affects the shape of the z-gradient in the region sampled by the OxyLite probe.

### 3.4. Uncertainty in Intracellular O_2_ Transport Mechanisms

Diffusion has historically been considered the primary driver of molecular transport within the cell [34]. However, intracellular convection has been observed in highly motile cells [35]. Since our model and experiments consider adherent cells over short timescales, motility is unlikely to play a role in generating intracellular convective currents. There is also some controversial evidence that intracellular thermal gradients may be large enough to create convective currents in motionless cells [36]. We would expect such currents to reduce the magnitude of intracellular O_2_ gradients. On the other hand, there is some contention over the effective rate of O_2_ diffusion in the densely packed intracellular environment. While our calculations here used ‘consensus’ values for this diffusion constant, if it were to be significantly lower, the model calculations would yield larger intracellular gradients. For example, if cytosol were significantly less permeable to O_2_ than extracellular/interstitial paths, that heterogeneity would make bulk measurement of packed cell diffusion rates more a measure of interstices than cytosol. O_2_ diffusion on the nanoscale may, thus, differ from that seen across millimeters. The model here should be considered a basic framework for understanding intracellular O_2_ gradients that can arise in the diffusion-dominated regime. In the event that future work develops a more detailed understanding of intracellular O_2_ transport parameters, the model will need to be amended.

Due to the computational demands of complex geometries, this model also only considers one-quarter of a cell in a uniform square grid. Acknowledging that cell seeding cannot be perfectly uniform, the average conditions experienced by cells may differ in clusters. It is likely that local conditions vary widely from this average case, either within clusters or more complicated geometries. The data shown in Figure 4 show that, well below confluence, sparsely spaced cells do not create significantly different gradients, but closer spacings or heterogenous cells are likely to produce different gradients.

## 4. Materials and Methods

### 4.1. FLIM-Based Oxygen Probes

Plasmids containing the untargeted myoglobin-mCherry (cytosolic Myo-mCherry), myoglobin-mCherry fused to the TFAM mitochondrial targeting sequence (MTS) (mtMyo-mCherry), myoglobin-mCherry targeted to the lysosomes (Lyso-Myo-mCherry), and myoglobin-mCherry fused to the RP2 plasma membrane targeting sequence (N-PM-Gly3-Myo-mCherry) were made for the intracellular O_2_ measurements. The untagged form of mCherry (alone) with no myoglobin or its fused form to mitochondria MTS (mt-mCherry) served as O_2_ insensitive controls for FLIM measurements [24]. The localization of mtMyo-mCherry and Lyso-Myo-mCherry was confirmed by mitoTracker Green and LysoView 488, respectively.

### 4.2. Cell Transfection with O_2_ Probes

HeLa cells were plated in eight-well chamber microscope slides (Ibidi GmbH, Martinsried, Germany) at a density of 2 × 10^4^ cells/cm^2^ in Modified Eagle Medium (DMEM, Gibco, Grand Island, NY, USA) with 10% fetal bovine serum (FBS, Mediatech Inc., Manassas, VA, USA) and 1% penicillin–streptomycin (Mediatech Inc., Manassas, VA, USA). Prior to transfection, the cells were placed into DMEM containing 20% FBS and antibiotics. The transfection medium was prepared by diluting 10 μg of the appropriate plasmid DNA and 20 μL of P3000 reagent (Invitrogen, Carlsbad, CA, USA) in 500 μL of Opti-MEM^®^ medium (Gibco, Thermo Fisher Scientific Inc., Grand Island, NY, USA). This was combined with a solution of 10 μL of Lipofectamine^®^ 3000 transfection reagent (Invitrogen, Carlsbad, CA, USA) in 500 μL of Opti-MEM^®^ medium and vortexed. The transfection medium was allowed to activate for 15 min before it was added to cells with a final plasmid amount of 2 µg of mtMyo-mCherry and Lyso-Myo-mCherry or 0.1 µg of Myo-mCherry and PM-Gly3-Myo-mCherry. The cells were incubated in this transfection medium for 24 h at 37 °C and 5% CO_2_, after which the cells were washed and covered with fresh cell medium containing 20% FBS as before. Cells were imaged 48 h after transfection.

### 4.3. Mitochondrial Modulator Treatment

Cells were treated with 2 μM rotenone and 2 μM antimycin A (AA) inhibitors or 50 μM 2,4-dinitrophenol (DNP) uncoupler for modulating the mitochondrial O_2_ consumption.

### 4.4. FLIM Setup

An Olympus IX81 confocal laser scanning microscope (Melville, NY, USA) equipped with a tunable Mai Tai BB DeepSee femtosecond laser (Spectra-Physics, Santa Clara, CA, USA) with wavelengths set to 780 nm was used for two-photon FLIM. The laser light was passed through a 690 nm dichroic mirror and directed to an Olympus UPLANSAPO 60×, 1.2 NA water immersion objective. A 675 nm short pass and a 647/57 nm bandpass filter (both from Semrock BrightLine^®^, Rochester, NY, USA) were used to reduce scattered light and to filter the mCherry signal, respectively. The fluorescence was detected using a PMC100 cooled detector (Becker & Hickl GmbH, Berlin, Germany), and the electrical pulse output from the detector was directed into an SPC-150N photon counting card (Becker & Hickl). For time-correlated single photon counting (TCSPC), the signals were synchronized with the pulses from the laser. Then, synchronization with the pixel, line, and frame clock from the scanning unit of the microscope was used for image construction in TCSPC mode. The cells were excited with a laser power ≤18 mW and imaged for 40–80 s to accumulate an adequate number of photons per pixel and to avoid photobleaching and photocytotoxicity. Image size was set to 256 × 256 (pixels)^2^, and TCSPC histograms were collected with 256 channels.

A miniature cell incubator connected to a gas mixing system (CO_2_–O_2_–MI, Bioscience Tools, San Diego, CA, USA) was mounted onto the microscope stage to provide a suitable cellular environment during imaging. The incubator kept the temperature at 37 °C, and the gas mixing system delivered mixtures of N_2_, O_2_, and CO_2_ inside the chamber according to the inputs set by the user. FLIM recordings were performed at stable %O_2_ (v/v) of 18.6%, 10%, 5%, and 0.5% in the microscope incubator, where 0.5% is the lowest %O_2_ attainable by our system. Typically, the cell culture (in culture dishes with a ~3 mm layer of medium above cells, without lids), when monitored by a 250 μm diameter bare-fiber O_2_ sensor (NX-BF/O/E, Optronix Ltd., Oxford, UK) connected to an OxyLite 1 Channel monitor (Optronix Ltd., Oxford, UK), reaches a stable pO_2_ within 1 h. The medium-imposed external pO_2_ (in mmHg) was measured at the bottom of the petri dish with or without live cells present. FLIM and OxyLite measurements were performed in the same experimental runs (the same cells and petri dishes).

### 4.5. Fluorescence Microscopy

For 3D imaging of the mitochondria distribution and investigation of mitochondria/lysosomes contact sites, the cells were incubated in 100 nM MitoTracker Green FM 100 (Invitrogen) or transfected with mtMyoglobin-mCherry and labeled with 10 μg/mL LysoView^TM^ 488 (Biotium, Fremont, CA, USA) at 37 °C for 30 min, respectively. Cells were washed three times with prewarmed cell culture medium. The 3D images of the mitochondria were acquired using a Zeiss LSM 780 confocal microscope (Carl Zeiss, Germany) equipped with a 63× oil immersion objective. MitoTracker Green was excited by an Argon 488 nm laser, and the fluorescence was directed toward a Photomultiplier Tube (PMT). Then, the green signals were spectrally filtered by an adjustable bandpass filter in photon-counting mode set to 499–534 nm. The cross-correlations between the signals of mtMyoglobin-mCherry/LysoView 488 and lifetime imaging of mtMyo-mCherry were performed using the Olympus IX81 confocal laser scanning microscope with wavelengths set to 780 nm for the excitation of mCherry and 880 nm for the excitation of LysoView. The emissions were collected using a bandpass filter 520/60 nm for LysoView 488 and a bandpass filter 647/57 nm for mCherry.

### 4.6. FLIM Analysis and Intracellular Mapping of pO_2_

FLIM images were processed as previously described [23]. Briefly, the fluorescence lifetime decays of Myo-mCherry in the whole cell, mitochondria, lysosomes, or plasma membrane were obtained by a double-exponential decay model in SPCImage (Becker & Hickl) at optimized goodness of fit (χ^2^). The mean lifetime was obtained for each single image (via amplitude weighting for each pixel) and averaged across multiple cells (*n* > 30). Then, the resulting lifetime values (*τ*(pO_2_)) were plotted against the medium-imposed external pO_2_ and a hyperbolic curve was fit to the data using MATLAB R2020b (The MathWorks Inc., Natick, MA, USA):

(1) $$\;\tau \left({\mathrm{pO}}_{2}\right)=\left(\tau_{max}-0.96\right)\frac{{\mathrm{pO}}_{2}}{K+{\mathrm{pO}}_{2}}+0.96,$$

where *τ_max_* is the longest average lifetime for Myo-mCherry at normoxia (O_2_ = 18.6%), and *K* is a fitting parameter related to the affinity of myoglobin for O_2_. The *τ*(pO_2_) values for non-respiring cells (those treated with rotenone/antimycin) were used as a reference for the lifetime of the probe at the environmental level of pO_2_ present in solution. Rearranging Equation (1), fixing the *K* and *τ_max_* to the values obtained from the reference curve, the effective pO_2_ at each lifetime value was back-calculated for the respiring cells. Then, pseudocolor mappings of pO_2_ in the cytosolic and mitochondrial environments were obtained using MATLAB R2020b (The MathWorks Inc.) equipped with the Image Processing Toolbox. More detailed description can be found in our earlier work [23].

Please note that the hyperbolic binding formula above yields widely varying pO_2_ values where the slope is small, i.e., above 50 mmHg, where the myoglobin is fully saturated with O_2_. Thus, we truncate color coding images at 50 mmHg and above.

### 4.7. Statistical Analysis

For each condition, FLIM was recorded in at least 30 cells. Kruskal–Wallis or Mann–Whitney U tests were used to evaluate whether the values in the independent groups are significantly different from each other. Analyses were carried out using SPSS 14.0 (a subsidiary of IBM, Chicago, IL, USA) software and statistical significance was defined at *p* < 0.05 (95% confidence level).

### 4.8. COMSOL Simulation of Intracellular pO_2_ Gradients

We modeled O_2_ gradients using finite-element analyses performed with the COMSOL Multiphysics Chemical Reaction Engineering Module. One-quarter of a cell’s mitochondrial network was modeled in a column of medium as shown in Figure 2. Symmetric boundary conditions (as described below) were applied on the four sides of the column such that results generalized to a case of a uniform square monolayer of symmetric cells. Oxygen concentration was fixed at an applied level at the top of the medium column to represent exchange with the larger volume of medium where convection was presumably the dominant mechanism of gas exchange. According to the density of macromolecules and other solutes in the cytoplasm, a lower O_2_ diffusion rate was used for it than in the bulk medium, in line with experimental measurements and that used in other models [37,38,39,40,41,42].

A realistic model of 3D mitochondrial structure was generated from imaging of C2C12 mouse myoblasts cells treated with MitoTracker Green FM (Invitrogen) as described above, and imaged with a pixel size of 108 × 108 × 100 nm (xyz) (Figure S1). While O_2_ consumption rate can vary widely in different conditions, similarly measured C2C12 and HeLa cells have shown per-cell consumption values within 5% of one another [43]. Image stacks were deconvolved using Huygens SVI on the basis of five point-spread functions measured using 0.1 µm microspheres, fluorescent blue/green/orange/dark red (TetraSpeck^TM^, Invitrogen). A representative cell was chosen for developing the 3D model. Initial postprocessing to binary images was performed in ImageJ following established protocol [44]. Since deconvolved images still showed stretching in z due to the acquisition method, binaries were then converted into skeletons using the bwskel function in the MATLAB R2020b Image Processing Toolbox (The MathWorks Inc., Natick, MA, USA). Due to the computational demands of larger models, one-quarter of this skeletonized model was used.

Slicer 3D was used to convert binary skeletons into STL meshes [45]. Volume geometry was adjusted to the correct spacing of the microscope raster. The volume was then oversampled by a factor of approximately two in each dimension to improve the handling of narrow and curved regions. Anisotropy was enforced during oversampling for a final pixel size of 50 nm in each dimension. An Otsu threshold was used to determine initial segmentation of mitochondrial volumes. The subsequent steps were performed according to what was observed to produce a model that balanced the geometric complexity of the volume with the constraints of simplicity needed for simulation. The following morphological operations were performed: grown on margin by four pixels, sliced into nine pieces with yz planes to reduce connectivity (without which extensively connected pieces were too complex to simulate), opened by nine pixels, median-filtered by seven pixels, opened by seven pixels, removed islands smaller than 2500 pixels, and closed by three pixels.

The resulting STL mesh was loaded into COMSOL with automatically determined repair tolerance and boundary partitioning and then imported into the model geometry with a relative simplification tolerance of 0.01 and a defect removal factor of 1.02. To ensure smooth modeling of high convoluted structures, models were meshed with a minimum element size of 1 nm.

The finalized geometry was placed close to the lower boundary of the model described above. Similarly, radii of the eighth-ellipsoid section representing the boundary between cytoplasm and medium were chosen on the basis of the smallest size that fit the full mitochondrial network without generating sections too thin for successful meshing: 30 × 30 × 12 μm (xyz). Unit model cell sizes of 35, 50, 100, and 150 μm correspond to center-to-center cell spacings of 70, 100, 200, and 300 μm or to cell seeding densities of 2.4 × 10^4^, 1 × 10^4^, 2.5 × 10^3^, and 1.1 × 10^3^ cells/cm^2^, respectively (in a uniform square grid). Gradients observed at spacings greater than 200 μm were not largely different than those seen at 200 μm spacing; thus, data from those models are not presented here, and larger spacings than 300 μm were not simulated.

Intracellular O_2_ transport was assumed to be governed by Fickian diffusion within all domains, with the addition of a term for consumption giving the following overall equation:

(2) $$\;\frac{\partial c}{\partial t}=D\nabla^{2}c+R,$$

where *c* represents the concentration, *D* is the diffusivity, and *R* is the volume consumption rate (all for O_2_) [46]. *R* was assumed to be nonzero only in mitochondria, and the diffusion coefficient and solubility parameters of the mitochondria were assumed to be equal to that of the cytosol [47]. Exact values of O_2_ solubility and diffusion in complex, solute-dense environments such as growth medium and even more so intracellular regions are contentious topics with limited experimental data [26]. These two sets of parameters were treated as the minimum, average, or maximum reliable literature values for the slow, standard, and fast diffusion conditions.

Symmetric boundaries were used to reduce the computational complexity of the model. The boundary condition applied to O_2_ concentrations at such boundaries was

(3) $$0=-n\cdot \left(-D\nabla^{2}c\right).$$

The O_2_ consumption rate in cells is highly dependent on coupling between metabolic pathways and cellular state. However, the overall pathway O_2_ consumption rate under steady-state conditions can be modeled as a hyperbolic, Michaelis–Menten style function of local O_2_ concentration:

(4) $$R=V_{\max}\frac{c}{K_{m}+c}$$

where *V*_max_ represents the maximum O_2_ consumption rate, and *K_m_* represents the apparent concentration at which the rate is half-maximal, which depend on the metabolic state and internal structure of the particular mitochondrion [48,49]. We modeled consumption of the form in Equation (4) with constant *V*_max_ and *K_m_* within a single mitochondrion as a volume reaction.

To set an upper bound on possible O_2_ consumption within a single mitochondrion, metabolic estimates based on respirometry of honeybee (*Apis mellifera*) flight muscles were used, as they are thought to represent maximal respiration rates [50,51,52]. Notably, these values exceed the greatest values estimated or measured for mammalian mitochondria by an order of magnitude. Simulations were also run with upper limit measured values from high-resolution respirometry of isolated mitochondria/muscle tissue and full body respiratory measurements, of which the latter exceed the former by a factor of three [53,54]. Simulations were performed with these extreme values for maximal consumption rate in order to determine the steepest gradients plausible. A factor in overall consumption rates in the cell is the total volume of mitochondria. While our imaging substantially captures the shape of mitochondrial networks in the cell, there is significant room for error and interpretation in determining the volume of those networks, and this may have led to our results under or overestimating total consumption of the cell.

Although diffusion across intracellular membranes is not thought to be a significant barrier to O_2_ diffusion compared to through aqueous environments [11], inward O_2_ flux across boundaries was modeled as

(5) $$\;P\left(c_{o}-\frac{S_{o}}{S_{i}}c_{i}\right)=-n\cdot \left(-P\nabla c_{i}\right),$$

where *c_o_* and *c_i_* represent the concentrations in the external and internal domains, respectively, *S_o_* and *S_i_* represent the solubilities in the external and internal domains, respectively, *n* represents the normal unit vector to the boundary, and *P* represents the boundary’s permeability to O_2_ [55]. The polymer bottom of the slide was treated as a thin boundary with mass transfer coefficient *k* between the cytosol and ambient air (assumed to be convectively maintained at constant pO_2_).

The incubator’s controlled atmospheric inflow was assumed to provide convective mixing and bulk flow to assure a relatively constant O_2_ tension. Hence, the O_2_ concentration at the upper simulated limit of the medium was constrained to the value predicted by Henry’s law in the applied atmosphere:

(6) $$c=S{\mathrm{pO}}_{2},$$

where pO_2_ is the partial pressure of O_2_ in the incubator, and *S* is the solubility of O_2_ in the medium. This applied O_2_ value may not correspond one-to-one with experimentally applied O_2_ values due to differences in medium thickness between experiments. Furthermore, assuming no convection and that O_2_ consumption is uniform across the surface of the slide, the majority of the O_2_ gradient is effectively 1D through the medium column until just above the consumption; hence, changing medium depth beyond a few factors of cell thickness is indistinguishable from changing the applied pO_2_.

In order to better understand how consumption differs in an environment with O_2_ supply from multiple directions instead of just from above (as is experienced by cells in suspension, for example), an alternative model with a layer of O_2_-permeable polydimethylsiloxane as the lower boundary was developed.

Lastly, since O_2_-permeable Ibidi culture slides were used for imaging, the lower boundary of the cell was considered an inflow of O_2_ per the manufacturer’s measured mass-transfer coefficient assuming that convection controlled the exterior [O_2_] to be the ambient concentration throughout. The values used in the model are shown in Table 3.

## 5. Conclusions

We investigated intracellular pO_2_ in HeLa cells with computational modeling and fluorescence lifetime imaging. The computational data quantitively agree with experimental results on O_2_ heterogeneity in the intracellular environment and provide insight into further predictions and impact of mitochondrial O_2_ consumption. Dimensional arguments and the measurement of diffusion in bulk tissue have long predicted the absence of significant intracellular O_2_ gradients; hence, we viewed our now-frequent observations of the same with a jaundiced eye. To date, however, we were unable to find fault with the observations themselves, as we systematically examined a variety of alternative explanations for the fluorescence lifetime changes seen, identifying no artifact yet. We observed, however, that the cell culture environment presents opportunities for low-dimension behavior; the cell that is ‘living with its back against an O_2_-impermeable wall’, especially at confluence, is clearly different from one in tissue.

## Figures and Tables

**Table 1:** Parameters of the hyperbolic fits. Parameter *K* was obtained from fitting the data presented in Figure 1a to Equation (1). *τ*_max_ is the longest average lifetime for Myo-mCherry measured at O_2_ = 18.6%. The imposed pO_2_ values were obtained from the OxyLite Pro fiber tip measurements.

	OxyLite Pro		
	*τ*_max_	*K*	*R* ^2^
Mitochondria	1.15 ± 0.03	6.889 ± 2.819	0.99
Cytosol	1.17 ± 0.04	6.852 ± 3.498	0.98
Rot/Antim	1.29 ± 0.03	6.764 ± 1.577	0.98

**Table 2:** Mean pO_2_ in mitochondrial and cytosolic cellular compartments for models with different applied O_2_ concentrations, cell spacings, and lower boundary conditions. Spacing describes the distance from the center of one cell to the center of the next cell in the square grid approximation of a monolayer.

O_2._ (%)	Spacing (µm)	Lower Boundary	Mean Mitochondrial pO_2_ (mmHg)	Mean Cytoplasmic pO_2_ (mmHg)	Difference (mmHg)
0.5	70	Glass	0.01	0.23	0.21
5			4.63	7.09	2.46
10			42.1	44.6	2.50
20			118.1	120.6	2.50
0.5	100	Glass	0.028	0.428	0.4
5			19.0	21.4	2.4
10			56.9	59.4	2.5
20			132.9	135.4	2.5
0.5	200	Glass	0.064	0.764	0.7
5			25.7	28.3	2.6
10			63.6	66.3	2.7
20			139.6	142.3	2.7
0.5	70	Ibidi Polymer	0.01	0.27	0.25
5			5.22	7.68	2.46
10			42.6	45.1	2.49
20			118.2	120.7	2.5
0.5	100	Ibidi Polymer	0.036	0.508	0.5
5			19.6	22.1	2.5
10			57.4	59.8	2.5
20			133.0	135.5	2.5
0.5	200	Ibidi Polymer	0.087	0.914	0.8
5			26.2	28.8	2.6
10			64.0	66.7	2.7
20			139.7	142.3	2.7
0.5	70	200 µm PDMS	0.38	1.27	0.89
5			33.2	34.5	1.31
10			71.2	72.5	1.32
20			147.2	148.5	1.32
0.5	100	200 µm PDMS	0.779	1.87	1.1
5			34.5	35.8	1.3
10			72.6	73.8	1.3
20			148.5	149.8	1.3
0.5	200	200 µm PDMS	1.08	2.30	1.2
5			35.0	36.4	1.4
10			73.0	74.4	1.4
20			149.0	150.4	1.4

**Table 3:** Parameters used for simulation.

Description	Symbol [Units]	Element	Value	References
O_2_ diffusivity ‘consensus’	*D* [cm^2^/s]	Medium,	1.9 × 10^−5^	[16,26,56,57,58]
		intracellular	0.9 × 10^−5^	[37,38,39,40,41,42]
		PDMS	5 × 10^−5^	[59]
O_2_ solubility	*S* [mol/cm^3^/mmHg]	Medium, intracellular	1.29 × 10^−3^	[16,38,60,61]
		PDMS	1.06 × 10^−2^	[59]
O_2_ permeability	*P* [cm/s]	Membrane	42	[11]
		PDMS	8	[62]
Slide mass transfer coefficient	*k* [mol/cm^2^/s/bar O_2_]	Slide	7.19 × 10^−12^	Ibidi manufacturer
Maximum O_2_ consumption rate	*V*_max_ [mol/m^3^/s]	Mitochondria	10	[37,38,43,50,63]
[O_2_] at half-maximal rate	*K_m_* [mol/m^3^]	Mitochondria	1 × 10^−4^	[26,58,64,65]

## Data Availability

Data are available on request due to privacy or other restrictions.

## Supplementary Materials

The supporting information can be downloaded at https://www.mdpi.com/article/10.3390/ijms232012597/s1.
