# Supplementary material for: Computational Modeling and Imaging of the Intracellular Oxygen Gradient
Source: Int J Mol Sci. 2022 Oct 20;23(20):12597. doi: 10.3390/ijms232012597 (PMC9604273; doi:10.3390/ijms232012597)
Supplement: Supplementary file 1 [file ijms-23-12597-s001.zip › ijms-1926698-supplementary.pdf]

International Journal of
Molecular Sciences

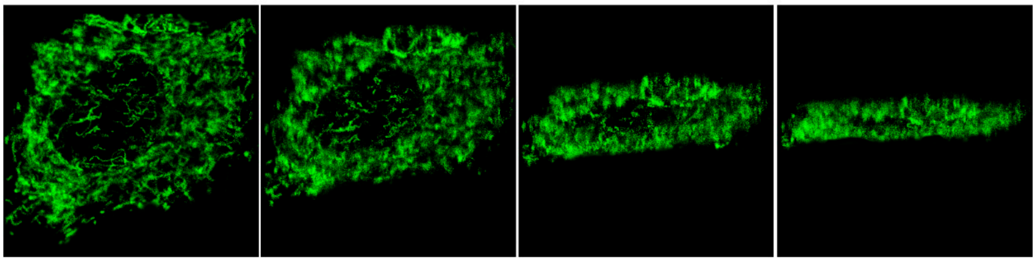

Figure S1. Z stack images of the mitochondrial structure of a C2C12 mouse myoblast cell used in the COMSOL model. Mitochondria was labelled with MitoTracker Green.

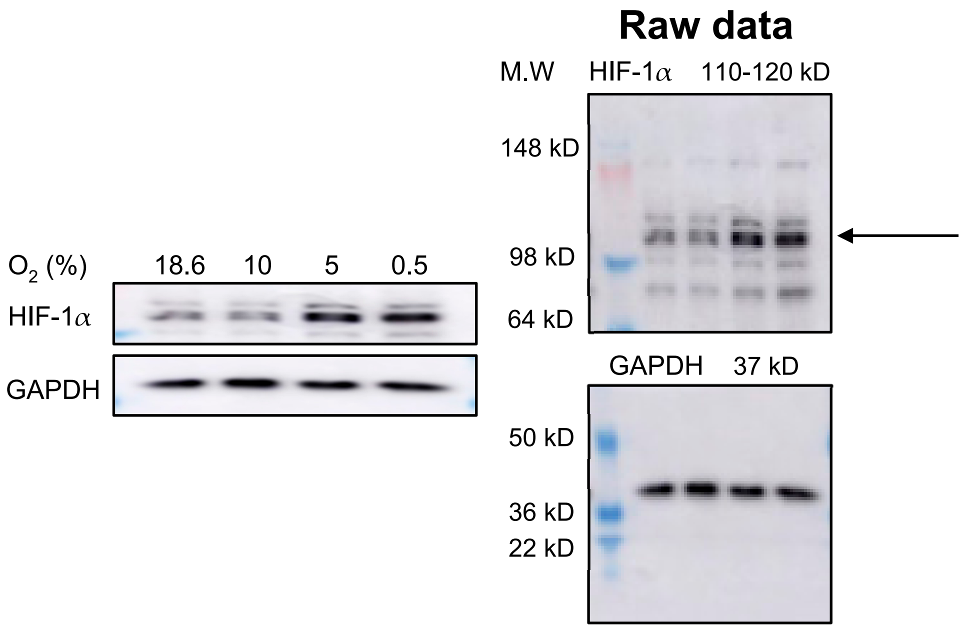

Figure S2. Detection of HIF-1α expression in the HeLa cells using immunoblotting. Cell pellets were lysed on ice for 20 min in RIPA buffer (50 mM Tris-HCl, pH 7.4, 0.15 M NaCl, 1.0 mM EDTA, 1% NP-40, 0.25% sodium deoxycholate) freshly supplemented with phosphatase and protease inhibitors (Millipore). Lysates were clarified by centrifugation at 14,000 rpm for 10 min. Proteins were quantified using the Coomassie plus protein assay (Thermo scientific, Waltham, MA, USA) and lysates were used immediately. The following antibodies were used: HIF-1α (Cayman Chemical, Ann Arbor, Michigan, USA) and GAPDH (Cell Signaling Technology, Danvers, Massachusetts, USA).

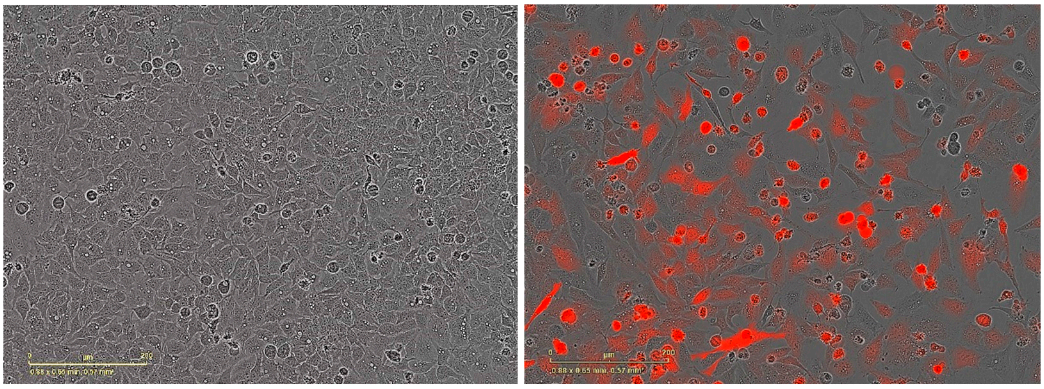

Figure S3. Phase contrast and fluorescence images of HeLa cells transfected with Myo-mCherry showing the cell confluency. This figure was taken and modified from the Supporting Information of the following article.

Penjweini, R.; Roarke, B.; Alspaugh, G.; Gevorgyan, A.; Andreoni, A.; Pasut, A.; Sackett, D.L.; Knutson, J.R. Single cell-based fluorescence lifetime imaging of intracellular oxygenation and metabolism. *Redox Biol*. **2020**, *34*, 101549. https://doi.org/10.1016/j.redox.2020.101549.

Table S1. Minimum pO_2 in mitochondrial and maximum pO_2 in cytosolic cellular compartments for models with different applied O_2 concentrations, cell spacings, and lower boundary conditions. Spacing describes the distance from the center of one cell to the center of the next cell in the square grid approximation of a monolayer.

O_2 (%)	Spacing (µm)	Lower Boundary	Min Mitochondrial pO_2 (mmHg)	Max Cytoplasmic pO_2 (mmHg)	Difference (mmHg)
0.5	70	Glass	0.00	0.56	0.56
5			2.84	10.17	7.33
10			40.27	47.80	7.53
20			116.23	123.73	7.49
0.5	100	Glass	0.00	1.13	1.13
5			17.11	25.09	7.98
10			55.05	63.06	8.01
20			131.04	139.05	8.01
0.5	200	Glass	0.00	2.06	2.06
5			23.69	32.58	8.89
10			61.66	70.57	8.91
20			137.65	146.60	8.95
0.5	70	Ibidi Polymer	0.00	0.68	0.68
5			3.43	10.78	7.35
10			40.77	48.25	7.48
20			116.39	123.98	7.58
0.5	100	Ibidi Polymer	0.00	1.35	1.35
5			17.70	25.69	7.99
10			55.48	63.53	8.05
20			131.13	139.15	8.02
0.5	200	Ibidi Polymer	0.00	2.46	2.46
5			24.23	33.38	9.15
10			62.04	71.22	9.18
20			137.70	146.81	9.11
0.5	70	200 µm PDMS	0.01	2.18	2.18
5			31.82	35.81	3.99
10			69.82	73.83	4.01
20			145.81	150.04	4.23
0.5	100	200 µm PDMS	0.04	2.90	2.86
5			33.28	36.99	3.71
10			71.28	75.02	3.74
20			147.28	151.20	3.92
0.5	200	200 µm PDMS	0.08	3.44	3.36
5			33.61	37.69	4.07
10			71.61	75.73	4.12
20			147.60	152.09	4.49
